# Microbiome Status of Cider-Apples, from Orchard to Processing, with a Special Focus on *Penicillium expansum* Occurrence and Patulin Contamination

**DOI:** 10.3390/jof7040244

**Published:** 2021-03-24

**Authors:** Reem Al Riachy, Caroline Strub, Noël Durand, Benjamin Guibert, Hugues Guichard, Florentin Constancias, Vincent Chochois, Félicie Lopez-Lauri, Angélique Fontana, Sabine Schorr-Galindo

**Affiliations:** 1 Qualisud, Univ Montpellier, CIRAD, Univ d’Avignon, Institut Agro, IRD, Univ de La Réunion, F-34398 Montpellier, France; noel.durand@cirad.fr (N.D.); benjamin.guibert@cirad.fr (B.G.); florentinconstancias@gmail.com (F.C.); vincent.chochois@cirad.fr (V.C.); felicie.lauri@univ-avignon.fr (F.L.-L.); angelique.fontana@umontpellier.fr (A.F.); sabine.galindo@umontpellier.fr (S.S.-G.); 2 CIRAD, UMR Qualisud, F-34398 Montpellier, France; 3 French Institute for Cider Production (IFPC), Domaine de la Motte, F-35653 Le Rheu, France; hugues.guichard@ifpc.eu

**Keywords:** cider-apple, microbiota, patulin, *Penicillium expansum*, blue mold disease

## Abstract

Patulin is a secondary metabolite produced primarily by the fungus *Penicillium expansum*, responsible for the blue mold disease on apples. It is found in apple products including apple cider when apple juice is added after fermentation. In the present study, two hundred and twenty-five cider-apples of the variety “Bedan”, cultivated in Brittany in France, were sampled from the orchard during harvesting until the storage step, right before processing. The patulin analysis on these samples reported a low contamination at the orchard and a significantly higher-level of contamination in the cider-apples starting from the transporting bin. The percentage of positive samples increased from 6% to 47% after 12 h in the harvesting bin before transporting and reached 95% after 24 h of transporting, decreasing then to 69% at the end of the storage. *Penicillium expansum* was quantified on the surface of apples using real-time PCR and was observed to be mostly consistent between the harvest and post-harvest steps. It was detected on average, on the surface of 85% of all sampled apples with a mean value around 2.35 × 10^6^ *Penicillium expansum* DNA/g of apple. Moreover, the changes in the fungal and bacterial epiphytic microbiota in the different steps were studied using a metabarcoding approach. The alpha and beta diversity analysis revealed the presence of unique and more diverse bacterial and fungal communities on the surface of apples picked from the orchard compared to the rest of the sampling steps. Potential indigenous biological control agents were identified on the surface of sampled apples. Future perspective includes developing actions of prevention and control of the contamination by *Penicillium expansum* during the harvest and along the various critical post-harvest stages before transformation in a sustainable development concern.

## 1. Introduction

“French cider” is a fermented alcoholic beverage made from apple juice, mainly produced in the northwest of France [1]. Depending on the level of sweetness related to the type of apple juices used, styles of cider are very diverse and vary from dry, with high alcohol content, to naturally sweeter ciders with a lower percentage of alcohol [2,3]. French cider is fruity, with strong bittersweet cider apple characteristics and an average alcohol content ranging from 2 to 4% (*v*/*v*) [3]. Cider production occurs in more than 25 countries around the world but its consumption is predominantly a European phenomenon, with west European countries accounting for 53% of the regional share of consumption in 2019 [3].

French apple cider is traditionally not regarded as a potentially hazardous food because of its high content in organic acids, mainly lactic acid (3–4 g/L), generating pH levels between 3.3–4.0. These acidity levels prevent the growth of bacterial pathogens such as *Salmonella* spp., *Escherichia coli* and *Staphylococcus aureus*, that could originate from the inert environment in contact with apples [4]. However, apple and non-fermented apple-delivered products can contain patulin, a mycotoxin produced by several filamentous fungi of the *Penicillium* genera. Among the species of this genera, *Penicillium expansum (P. expansum)* is the main producer of patulin and is considered an important pre-harvest and post-harvest contaminant of apples worldwide [5]. It is responsible for the blue mold decay, one of the most severe post-harvest diseases affecting apples. *P. expansum* spores can be found on the surface of healthy apples and can only grow and produce patulin by invading wounds of fresh apples caused by insect injuries, stem punctures and bruises created during picking and handling procedures [6,7]. Patulin is found at higher levels in lower grade or unfit apples destined for processing into juice, cider, sauces, jellies and purees [8]. However, patulin has been regularly detected in apple products made from externally healthy-looking apples which were internally colonized by *P. expansum* initiating from the opening calix tube during apple blooming [9]. Furthermore, once patulin is produced, practices like pasteurization can only marginally reduce patulin content in bottled apple juice and are only effective for the elimination of the fungus [10].

Patulin presents a major safety concern for apple products because of its severe acute and chronic effects in human beings. Acute patulin-toxicosis can lead to convulsions, agitation, edema, dyspnea, ulceration, intestinal inflammation, vomiting [11,12]. Chronic health risks of patulin ingestion may include genotoxic, neurotoxic, immunotoxic, immunosuppressive, teratogenic effects and severe damage to mammalian organs, especially kidneys and livers [13,14]. In comparison to the toxicity of other mycotoxins such as ochratoxin A, patulin has a higher immunotoxicity level [15]. The International Agency for Research on Cancer (IARC) classified patulin in group 3 “not classifiable as a carcinogen to humans”, although a study showed that long-term exposure of rats and mice to patulin indicated potential carcinogenicity of this mycotoxin [14]. Maximum permitted levels were established by the European Union (EU) ranging between 50, 25 and 10 µg patulin/kg, respectively for fruit juices, nectars and fermented apple beverages, solid apple products and apple-based products for infants and young children [16,17].

In apple cider, patulin is degraded during fermentation due to yeast action. A recent study showed that the level of patulin in contaminated musts was decreased six-fold after two days of fermentation [18]. Therefore, the presence of patulin in cider is mostly due to the adding of apple juice, in certain industries, in order to produce “sweet cider” or low-fermented cider [19]. However, the factors influencing the accumulation of patulin in apple-based products are still poorly understood and may result from a variety of interacting parameters, in particular in post-harvest processes.

Moreover, the epiphytic microbiome of fruits consists of a large number of microorganisms who play more or less an important role in maintaining health, quality and disease resistance of the fruit during pre and post-harvest steps. So far, most microbiome studies associated with apples have mainly focused on the evaluation of the microbial diversity at a single post-harvest point. However, to this day, little is known about the diversity of the epiphytic microbiome associated with apples used to produce cider. In fact, most research related to cider microbiome has concentrated on bacteria of oenological interest, like acetic and lactic acid bacteria present in the microflora in fermented cider and on pathogens. Few studies have investigated epiphytic bacteria and fungi on apples and were mainly focused on species involved in the fermentation process. Therefore, the study of cider-apple microbiome in post-harvest stages is a promising, under-used tool to better understand the phenomena regulating the contamination by *P. expansum* and patulin production.

In this study, culture-dependent and independent (metabarcoding) methods were combined to determine the composition of epiphytic bacterial and fungal communities present on the cider-apples in post-harvest stages. One of the main advantages of metabarcoding approaches is the ability to rapidly and accurately identify the effect of post-harvest practices on the microbial diversity [20]. In parallel, patulin content in the sampled cider-apples was determined by HPLC-MS/MS and *P. expansum* was quantified on the surface of apples using real-time PCR in order to identify critical post-harvest points of contamination. Finally, potential indigenous biological control agents were identified on the surface of sampled apples at the different post-harvest steps.

## 2. Materials and Methods

### 2.1. General Overview of the Apple Sampling and Handling Processes

**Figure 1:**
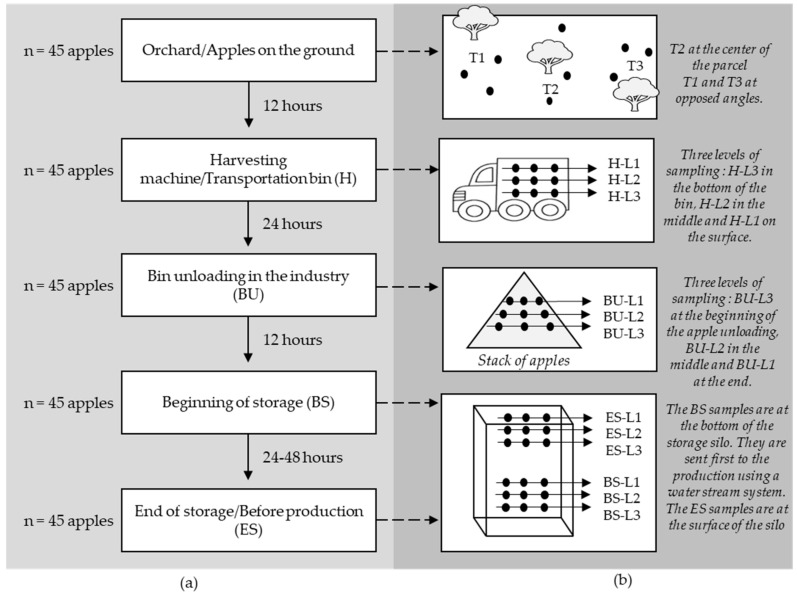
(**a**) Flow diagram of the different sampling steps of the cider processing chain with the number of apples sampled and the period between two consecutive steps. (**b**) Sketches of each sampling step with an illustration of the general location of selected apples. Each black dot represents a sample composed of fives apples picked randomly. The sketches are just for illustration purposes and do not reflect the exact location of the sample.

The aim of this study is to characterize the composition and dynamics of the microbial ecosystem while focusing on the *P. expansum* and patulin contaminations in apples. The sampled apples are selected throughout different steps of the apple-cider production chain, starting from mechanically harvested apples until the unloading of apples in the industry right before processing. The apples are of the variety “Bedan”, a traditional French bittersweet cider variety, and were cultivated in Brittany, France. The same batch of apples was followed up from the orchard till the industry. In the orchard, the apples were subjected to an insecticide treatment against the codling moth, a fungicide treatment against apple scab and finally a treatment against the post-harvest black rot and phytophthora. This is the minimum treatment applied in conventional cider apple orchards. Even if it has an effect on the microbiome of the harvested apples, it will still be possible to assess the impact of the post-harvest stages on its evolution, which is the main objective of this study. The fruits were mechanically harvested using an appropriate machine to shake the trees and collect the apples from the ground. The fruits were then transported in another bin within 12 to 24 h to the processing industry where they were stored outside in open concrete silo before processing. The sampling was done randomly and the apples were selected at the different sampling steps without taking into consideration their health status. Thus, damaged apples, if present, were not removed during analysis, in order to avoid influencing the results. In total, 225 apples were collected by hand using sterilized gloves and placed separately in sterilized plastic bags. Figure 1 presents the flow diagram of the different sampling steps with a basic description of the location and number of sampled apples and the conditions of each step. At reception, the apples were visually inspected and were given initial numerical scores from 1 to 5 representing the level of damage of each apple (1 representing the least spoilage and 5 the most). All the samples were then kept at 3 °C until further analysis. The apples originating from the same sampling step were either treated individually or in a group of five or in a group of 15. The pooling of the apples into groups of five and 15 was conducted randomly without taking into consideration the health status of the apples. In order to ensure representivity in the analysis of the entire bin and storage silos, the pooling was done as follows: five apples were randomly selected from each level to be treated individually, five other apples were selected randomly at each level and pooled together in groups of five, and finally the five remaining apples at each level were pooled together in groups of 15 with the five from the two other levels. The different levels established at each sampling stage are illustrated in Figure 1. This enabled the comparison between different treatment modalities in order to determine whether these differences have an impact on the obtained results or not.

For all the following tests, the statistical significance of the differences in samples treated individually, in a group of 5 or in a group of 15 were tested by one-way ANOVA followed by Tukey’s honestly significant difference test (Tukey’s HSD, *p* < 0.05) using Statistica V.13 software (Statsoft Inc., Tulsa, OK, USA). No significant differences were observed between the different treatment modalities. Therefore, the results are presented without the consideration of the different treatment modalities (individually, in groups of five and groups of 15). In case the apples were treated by group of five or group of 15, the mean was calculated and, in all cases, the results were expressed per g of apple.

### 2.2. Culture Dependent (Plating) Analysis of the Apple Surface Microbiota

The apples were placed in sterile polyethylene bags, containing 50 mL of washing solution per apple. Washing solution was composed with 0.15 M NaCl supplemented with 0.1% Tween20 (*v*/*v*) in distilled water. Apples were manually scrubbed against the bag for approximatively 5 min. 25 mL of the resulting washing solution was centrifuged at 4500× *g* for 10 min at room temperature. The other half of the washing solution was stored at 4 °C to be used later for DNA extraction. The pellet obtained after centrifugation was resuspended in 5 mL of sterile peptone water (Biokar Diagnostics, Allone, France), to which a 3 serial decimal dilution was performed in 9 mL of sterile saline water. Then, 0.1 mL of the stock solution and of each of the 3 serial diluted solutions were used to inoculate, in duplicate, 3 different culture media. Potato Dextrose Agar (PDA 3.5) (Biokar Diagnostics, Allone, France) supplemented with tartaric acid was used for the cultivation and isolation of mold strains, Plate Count Agar (PCA) (Biokar Diagnostics, Allone, France) for the isolation of bacterial strains and finally the Yeast Extract Glucose Chloramphenicol Agar (YEGC) (Merck, Darmstadt, Germany) for the isolation of the yeast strains. The Petri dishes were incubated respectively at 25 °C (PDA 3.5) and at 30 °C (YEGC and PCA) for 3 days to allow macroscopic differentiation between molds and yeasts [21,22]. After that, the Petri dishes were removed and the viable cells were counted. The two successive dilutions retained for colonies counting are those whose dishes represent a significant number of colonies (between 15 and 300 for bacteria and 15 and 150 for yeasts and molds). In cases of uncertainty, microscopic observation was made in order to confirm the identification. The results were presented with confidence limits of 95%.

### 2.3. Extraction and Analysis of Patulin in Apples

The apples were pressed using a laboratory paddle blender (Stomacher^®^ 400, Seward, England, UK) in sterile conditions. About 10 g of apple puree of each sample (no replicates) were weighed into a Falcon flask of 50 mL to which 150 µL of pectinase from *Aspergillus aculeatus* enzyme solution (SIGMA, Darmstadt, Germany) were added followed by 10 mL of H_2_O [23]. The pectinase was used on the apple puree in order to maximize the recovery of the patulin because these small particles are often retained in the pectin net. Then, the mixture was homogenized and retained overnight at room temperature. The samples were then centrifuged at 4500× *g* for 10 min and the supernatant was pipetted into a 50 mL Falcon flask to which 2 g of sodium bicarbonate and 15 g of anhydrous sodium sulfate were added. Ten mL of the solvent, which consists of a mixture of ethyl acetate/hexane (60/40, *v*/*v*), were then added. The mixture was thoroughly agitated for 10 min on a rotary agitator at 300 rpm and then centrifuged at room temperature at 3000 rpm for 10 min. 10 mL of the supernatant were collected in a 15 mL Falcon flask and evaporated in a SpeedVac Concentrator Plus (Eppendorf, Hamburg, Germany) for 60 min at 30 °C. The dry extract is suspended in 1 mL of water acidulated (0.5% acetic acid) (*v*/*v*) and the samples were then sonicated for 30 min for better dissolution. The samples were filtered through a 0.45 µm cellulose acetate (CA) syringe filter (Phenomenex, California, USA) into a clean 2 mL vial. In order to determine the concentration of patulin in the different samples, 100 µL aliquots were injected onto the HPLC system (SHIMADZU, Kyoto, Japan). The detection of the patulin was made through a lichrospher 5 µ ODS 250 × 4.6 mm C18 HPLC Column at a flow rate of 1 mL/min at a temperature of 35 °C. The mobile phase consists of ultra-pure water (phase A) and acetonitrile (Phase B). A mobile phase gradient program was started at 95% A (0.01 min), 98% A at 16 min, 40% A at 20 min, 95% A at 26 min until 30 min. The presence of patulin was detected at a 277 nm wavelength. A calibration curve was realized with patulin standard (LIBIOS, Vindry-sur-Turdine, France) using concentrations ranging from 1.5 ng/mL to 250 ng/mL. Finally, the LOD and LOQ of the method were calculated and the values were determined as follows: LOQ: 1.5 ng/mL and LOD: 0.5 ng/mL.

### 2.4. DNA Isolation from the Surface of the Apples

The DNA was extracted from 25 mL of the washing water using the FastDNA™ SPIN Kit (MP Biomedicals, Illkirch, France) with a FastPrep^®^ Instrument to lyse the DNA. The washing water was centrifuged at 4500× *g* for 10 min and the pellet was resuspended in 1 mL in the lysing solution CLS-TC (MP Biomedicals) and then transferred to the Lysing matrix A tube which is a polypropylene tube containing garnet matrix and one 1/4” ceramic sphere. The samples were then ground using the FastPrep^®^ Instrument for 50 s at 6 m/s. The rest of the extraction was made as specified by the manufacturer. Lastly, the DNA was suspended in 50 µL of DNase free water (DES). The DNA purity ratio and concentration were then measured using a Nanodrop ND 8000 spectrophotometer (Thermo Fisher Scientific, Waltham, MA, USA).

### 2.5. Quantification of P. expansum on the Surface of Apples Using Real-Time PCR

In order to determine the *P. expansum* abundance on the surface of apples, q-PCRs were conducted with the set of primers Pexp_patF_F/Pexp_patF_R (0.2 µM, Sigma-Aldrich) designed from the patF gene, involved in patulin biosynthesis [24]. These primers amplified a PCR product of 92 bp and their sequences are shown in Table 1. All the DNA samples were diluted at (1:5, *v*/*v*) in DNAse free distilled water. The q-PCR reactions were carried out in 384 well-plates, prepared using the Echo^®^525 Liquid Handler (LABCYTE, San Jose, CA, USA) and each sample was analyzed in triplicate. The q-PCR reactions were performed in the LightCycler^®^ 480 Real-Time PCR System (Roche Applied Science, Mannheim, Germany) using the following PCR thermal cycling conditions: 95 °C for 20 s, 45 cycles of 95 °C for 30 s, 63 °C for 30 s and 72° for 15 s. Following the final amplification cycle, a melting curve was constructed by heating at 95 °C for 5 s followed by 65 °C for 1 min and then a cooling step at 40 °C for 30 s. The PCR reactions were performed in a total volume of 14.5 µL consisting of 10.5 µL of SensiFAST SYBR^®^ No-ROX (Bioline, Paris, France), 0.42 µL of the two primers (10 µM), PCR-grade water and 0.5 µL template DNA. For each 384 well-plate prepared, ten-fold dilutions of *P. expansum* (NRRL 35695, Northern Regional Research Laboratory, Peoria, IL, USA) pure genomic DNA whose concentration was previously determined were added in order to generate a standard curve that allows the quantification of *P. expansum* DNA on the surface of apples. The LOD and LOQ of the method were calculated and the values were determined as follows: LOQ: 10^4^ *P. expansum* DNA/g of apple and LOD: 10^3^ *P. expansum* DNA/g of apple. The quantification values of the DNA and the threshold cycle (Ct) values were automatically determined by the LightCycler^®^ 480 system software. A negative control containing DNAse free distilled instead of the DNA sample was added in every plate to exclude or detect any possible DNA contamination.

### 2.6. DNA Amplification and Sequencing

#### 2.6.1. Library Construction and Sequencing

DNA samples were quantified using a spectrophotometer-Nanodrop ND 8000 (Thermo Fisher Scientific) and the total DNA concentration was adjusted to 15 ng.µL^−1^. The fungal ITS2 region of ribosomal DNA was amplified using the set of primers ITS86 and ITS4 [25]. The bacterial 16S V3/V4 region was amplified using the primers 341F and 785R and a pair of peptide-nucleic-acids PCR blockers (PNA Bio Inc, Newbury Park, CA, USA) was added in order to reduce the generation of undesired chloroplast and mitochondrial amplicons. The sequences of both sets of primers are shown in Table 1. Those primers were modified to include Illumina adapters (www.illumina.com, accessed on 16 February 2021) for subsequent multiplexing. The ITS2 PCR was performed in a total volume of 25 µL containing 12.5 µL of AmpliTaq Gold™ 360 Master Mix (Thermo Fisher Scientific), 0.625 µL of each primer (0.25 µM), PCR-grade water and 5 µL template DNA (no replicates). The reactions were incubated in a Mastercycler X50 (Eppendorf France SAS, Montesson, France) under the following cycling conditions: 94 °C for 10 min, 30 cycles of 95 °C for 30 s, 55 °C for 45 s, 72 °C for 30 s and a final elongation at 72 °C for 10 min. PCR for 16S rRNA gene amplification was performed in a total volume of 25 µL containing 12.5 µL of AmpliTaq Gold™ 360 Master Mix (Thermo Fisher Scientific), 0.625 µL of each primer (0.25 µM), 1 µL of each PNA (0.5 µM), PCR-grade water and 5 µL template DNA (no replicates) under the following cycle conditions: 95 °C for 5 min, 30 cycles of 96 °C for 1 min, 78 °C for 5 s for PNA clamping, 54 °C for 1 min, 74 °C for 1 min and a final elongation at 72 °C for 10 min. After a magnetic bead purification (Clean PCR, Proteigene, Saint-Marcel, France), the indexing PCR was performed in a total volume of 18 µL (5 µL of first round PCR products, 9 µL Phusion^®^ High-Fidelity PCR Master Mix (NEB, Evry, France), 2 µL I5 index-adapter, 2 µL I7 index-adapter). Cycling conditions: 95 °C for 3 min then 10 cycles of 95 °C 30 s, 55 °C 30 s, 72 °C 30 s then final elongation of 5 min at 72 °C. A set of 384 in-house index pairs was used to be able of multiplexing the whole samples on a single MiSeq run. After purification with magnetic beads, these final PCR products were pooled and paired-end sequenced on a MiSeq Illumina sequencer using MiSeq Reagent Kit v3 (600-cycle; Illumina, San Diego, CA, USA).

#### 2.6.2. Data Analysis and Statistics

Sequencing data were demultiplexed, trimmed using cutadapt. Forward and reverse paired-end reads were then merged, cleaned from chimeras, filtered and assigned a taxonomy using a dada2 based workflow. The taxonomy assignment databases used were UNITE 8.2 [26] and RefSeq_RDP 16S databases for ITS and ADNr 16S, respectively. Alpha and Beta diversity population structure and composition were analyzed using the phyloseq package (version 1.32.0) in R (version 4.0.0) after rarefaction to an even number of reads of 5516 and 10,032 per sample for 16S and ITS2 respectively. Statistical analyses were carried out in R. Alpha diversity was compared between sampling steps using Tukey’s HSD pairwise comparison (α ≤ 0.05). Beta diversity differences between sampling steps was tested using permutational ANOVA (PERMANOVA, adonis function) with 9999 permutations.

## 3. Results

### 3.1. Culture Dependent (Plating) Analysis of the Apple Surface Microbiota

**Figure 2:**
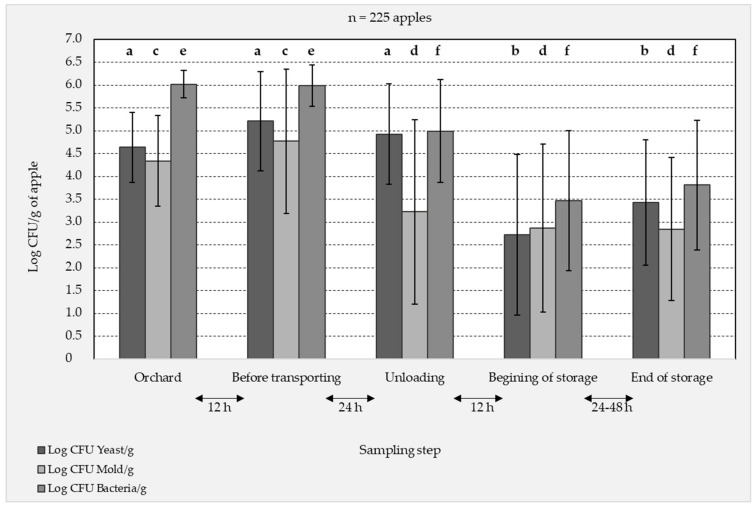
Culturable bacteria, yeast and mold populations on the surface of apples collected at different steps of the apple cider production chain before transformation into juice. The results are expressed as log_10_ CFU/g of apple, determined by plate counting on three different growth media. The values are the average of a duplicate experiment of 27 apple samples collected at the orchard and 19 apple samples collected at the four final steps (including apples treated individually, by group of 5 and by group of 15), total number of apples analyzed = 225. The different letters denote homogeneous groups revealed by post-hoc tests (*p* ≤ 0.05) done for the culturable populations of each microbial type throughout the different sampling steps.

The bacterial and fungal compositions at the surface of the cider-apples were analyzed by a culture-dependent approach (plating). Three different growth media, favorable for the growth of bacteria, yeast or mold were inoculated, in duplicate, with the apple washing water and its 3-fold decimal serial dilution. Figure 2 shows the follow-up of the yeast, mold and bacteria population counts on the surface of 225 apples at the different sampling steps. Overall, it is observed that the different microbial types were present on the surface of apples on average at 10^5^ CFU/g of apple. Higher standard deviation values were observed in the unloading and storage steps than in the first two steps, indicating a high variability among the apple samples in the steps following the transport. The bacteria population counts for all samples were consistently higher than those of molds and yeasts with high numbers of culturable bacteria ranging from 10^7^ CFU/g of apple in the orchard to 10^5^ log CFU/g of apple in the storage silo. The last three process steps significantly reduced bacteria and mold plate counts by two orders (one-way ANOVA followed by Tukey’s HSD, *p* < 0.05). The transportation step had an important effect on both mold and bacteria populations. However, the yeast population was more resistant to the changes occurring during the transportation and the culturable population counts were similar in the first three steps and significantly decreased in the storage silo.

### 3.2. Quantification of Patulin Content in Apples by HPLC-MS/MS

**Figure 3:**
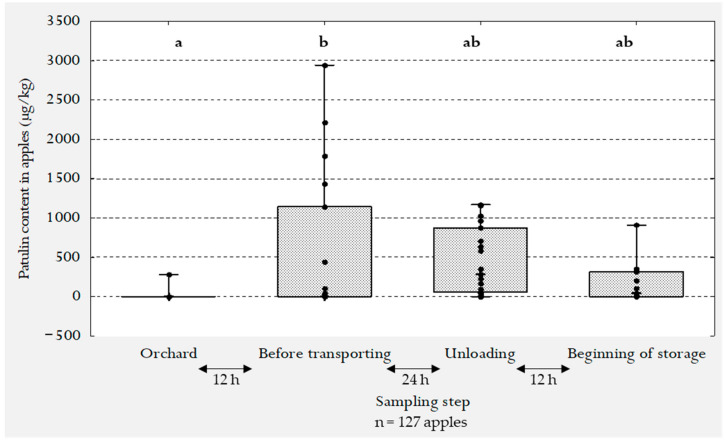
The distribution of patulin (µg/kg) at different post-harvest steps in total of 127 sampled cider-apples. The upper and lower ends of the whiskers represent respectively the minimum and the maximum values of patulin analyzed at each step. The black dots illustrate the distribution of patulin among all samples showing a wide distribution in the step before transporting that is narrowed at the last two steps.

The sampled apples were analyzed for their patulin content. Table 2 presents the sample sizes per step with the percentage of positive samples (including individual apples, groups of five apples and groups of 15 apples) where patulin was quantified (µg/kg of apple). Patulin was found in one sample out of 16 (6%) at the orchard at a level of 208.8 µg/kg. The percentage of positive samples increased to 47% after 12 h in the harvesting bin before transporting. This difference was statistically significant (according to non-parametric Kruskal–Wallis/FDR, *p* < 0.001). A wide variation of the results between the different samples in the bin was observed in Figure 3 with one sample showing a high level of patulin (2943.1 µg/kg). These data highlight the general sampling problems associated with mycotoxin analyses and the heterogeneous distribution of the mycotoxin within the different batches. Furthermore, the sampled apples in the bin sustained shocks more or less severe due to the harvest machine explaining the high variability of patulin content in the different samples. After 24 h of transporting, patulin was detected in 95% of the analyzed samples with levels ranging from 39 to 1168 µg/kg (Table 2). Although there appeared to be an overall decrease in the patulin levels in these latter samples and in those sampled at the beginning of storage, the decrease was not shown to be statistically significant (Kruskal–Wallis/FDR, *p* > 0.05). This was most probably due to the wide variation in the patulin levels found in the harvesting bin apples. There were no significant differences in the level of patulin between apples sampled during the unloading and the beginning of storage. In these latter samples, 69% were tested positive with a patulin level ranging from 24 to 356 µg/kg. Finally, no significant differences were observed between the orchard step and the two final steps (Table 2). The patulin contamination levels were significantly affected by the mechanical harvest of apples and the conditions of the post-harvest steps.

### 3.3. Quantification of P. expansum on the Surface of Apples Using Real-Time PCR

**Figure 4:**
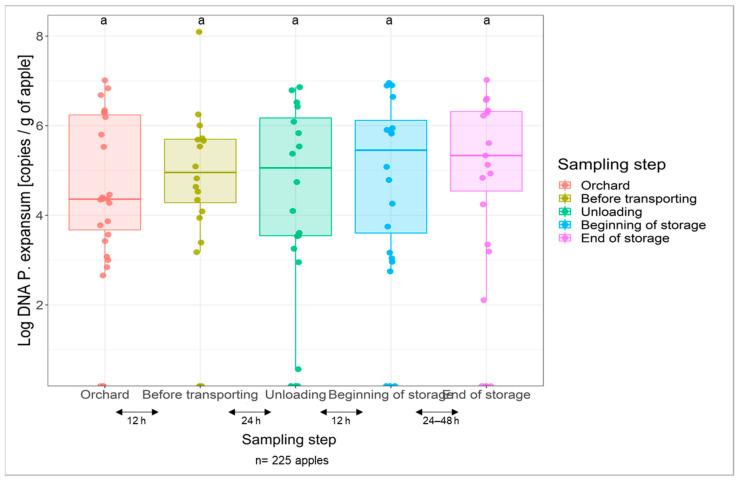
Boxplot illustrating the DNA amounts of *P. expansum* per g of apple on the surface of 225 cider-apples sampled at the orchard and at different post-harvest steps. The fungal abundance within each step was measured in three replicates by qPCR. *P. expansum* is present on the surface of apples at the orchard and is maintained throughout the post-harvest steps. One-way ANOVA test showed no significant difference for fungal biomass at the sampling stages.

DNA amounts of *P. expansum* per gram of apple were measured by qPCR at the different sampling steps (Figure 4). *P. expansum* abundances were observed to be mostly consistent between the harvest and post-harvest steps with a mean value around 2.35 × 10^6^ *P. expansum* DNA/g of apple. One-way ANOVA test showed no significant differences between the *P. expansum* abundance mean values recorded per step (*p* > 0.05). As shown in Table 3, the fungal *P. expansum* DNA was detected on the surface of 88,46% of the ground-picked sampled apples at the orchard level with amounts ranging from non-detectable to 1.04 × 10^7^ *P. epansum* DNA/g of apple and a mean value of 1.19 × 10^6^ *P. expansum* DNA/g of apple. For the rest of the steps, *P. expansum* DNA was detected on 84.21% (16 positive samples out of 19 in each step) of the total sampled apples with mean values ranging between 1.22 × 10^6^ *Pe* DNA/g of apple and 7.13 × 10^6^ *Pe* DNA/g of apple. Figure 4 also shows a high dispersion of the *P. expansum* DNA values across samples in the steps that follow the transportation especially on apples selected at the end of the storage step with a slight increase in *P. expansum* DNA amounts (<96 h after harvest) i.e., right before being carried by conveying water to the processing line. Figure 3 shows a very limited production of patulin by the apples sampled in the orchard (detected in 6% of tested samples), however, the amount of *P. expansum* DNA detected at the surface of the same apples at this step was important (88.46% of sampled apples) (Figure 4). Moreover, the *P. expansum* DNA abundance was steady for the rest of the process steps.

### 3.4. General Structure of the Bacterial and Fungal Microbiota of the Surface of Cider-Apple

#### 3.4.1. Sequencing Results

After paired-end alignments, quality filtering and deletion of chimeras, singletons, and mitochondrial and chloroplast sequences, a total of 1,609,179 bacterial 16S rRNA and 3,032,303 fungal internal transcribed spacer (ITS) reads were recovered and assigned to respectively 1066 bacterial and 3978 fungal amplicon sequence variants (ASVs) in a total of 101 samples. Sample heterogeneity was removed by rarefying to an even depth of 10,032 (ITS) and 5516 (16S) reads per sample, i.e., samples with less than 10,000 and 5000 reads were removed respectively for the ITS and 16S analysis. The rarefaction curves tended towards saturation, illustrating that the current sequencing depth was sufficient for both fungal and bacterial diversity analysis. The number of ASVs reflects only the washed-off epiphytic community and not the entire host microbiome. The analysis of the rarefied ASV table reported an important level of variability between the different samples with the highest diversity reported on the surface of apples sampled at the orchard.

#### 3.4.2. Epiphytic Fungal and Bacterial Microbiota of Cider-Apples

A total of 187 identified bacterial genera belonging to 12 phyla, 25 classes, 30 orders and 82 families were detected on the surface of the cider-apple samples. Bacterial ASVs, across all samples, were assigned to *Proteobacteria* (66.17%), *Firmicutes* (25.47%), *Actinobacteria* (6.92%), *Bacteroidetes* (0.64%), *Acidobacteria* (0.28%) and *Chlamydiae* (0.26%). Other phyla were detected but at low frequency such as *Cyanobacteria*, *Deinococcus*, *Planctomycetes*, *Verrucomicrobia* and other unidentified bacteria. The *Proteobacteria* were mainly represented by the class *Alphaproteobacteria* (58.64%) and *Gammaproteobacteria* (5.36%), and *Firmicutes* were represented primarily by *Bacilli* (25.32%) and *Clostridia* (0.13%), *Negativicutes* were also detected but at very low frequency. The class *Alphaproteobacteria* were largely represented by the family *Acidobacteria* and the class *Bacilli* contained mostly *Bacillaceae* in the orchard and *Paenibacilliaceae* in the rest of the sampling steps.

#### 3.4.3. Compositional Differences in the Diversity and the Taxonomy of Fungal and Bacterial Microbiota Induced by the Different Sampling Steps

**Figure 5:**
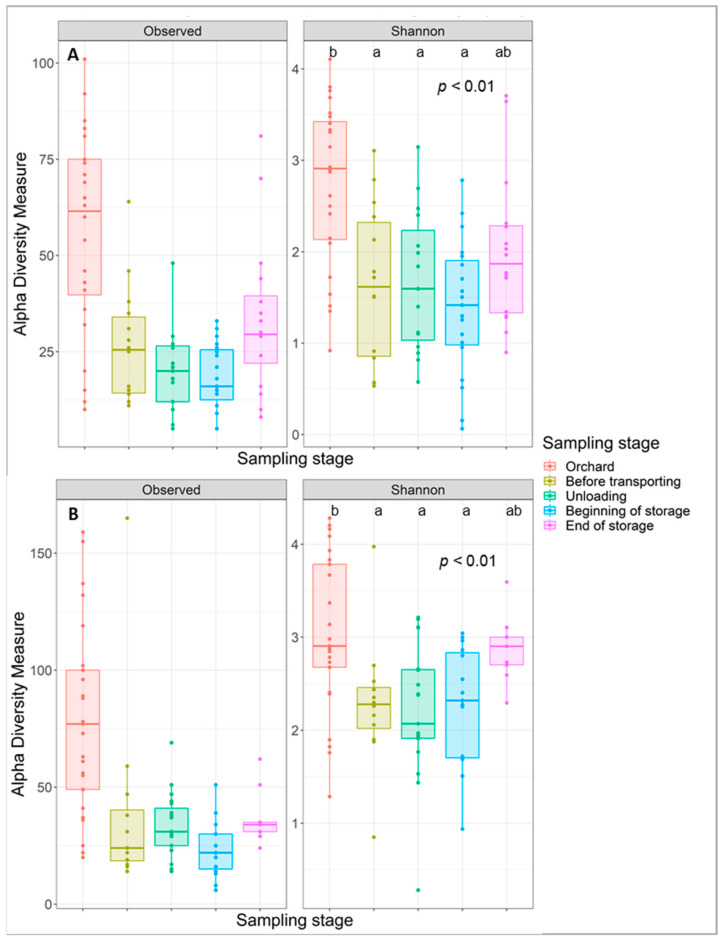
Boxplots illustrating the differences in the observed and Shannon diversity measures of the fungal (**A**) and bacterial (**B**) communities on the surface of the tested apples sampled at the different post-harvest stages.

**Figure 6:**
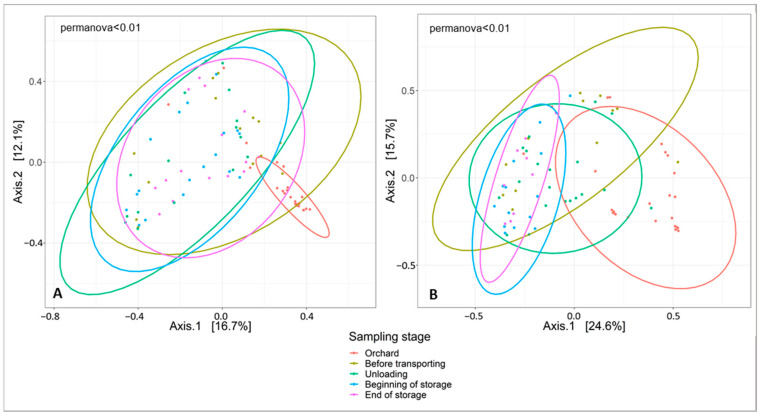
Principal coordinate analysis (PCoA) of fungal (**A**) and bacterial (**B**) populations associated to the surface of cider-apples sampled at the five post-harvest stages based on the beta diversity metric Bray Curtis.

The fungal and bacterial diversity within the cider-apple samples was assessed by Shannon diversity index (Figure 5A,B). The ANOVA performed on the bacterial and fungal alpha diversity data show a significant difference across samples for the sampling stage variable (*p* < 0.001). The bacterial and fungal total numbers of taxa were consistently higher in the orchard than in the rest of the sampling steps. The changes in fungal and bacterial communities due to the different steps of the apple-cider production chain were tested by comparative analyses. The results of alpha diversity showed that the apples picked from the orchard share a more diverse fungal and bacterial microbiota than the apples sampled in the transporting bin, during unloading and at storage step (Figure 5). The existence of significant differences between the different fungal and bacterial microbiomes was confirmed by the analysis of beta diversity, applied on the whole bacterial and fungal dataset. Fungal and bacterial beta diversity communities among samples were determined by Bray-Curtis distances (Bray-Curtis PCoA) illustrated in Figure 6 (Figure 6A,B, respectively). The PCoA showed that samples from the orchard exhibited differences in fungal and bacterial communities compared to those picked from the rest of the sampling steps, meaning that the mechanical harvest of apples followed by a waiting period of 12–24 h in the transporting bin, had a significant effect on the epiphytic microflora of cider-apples (PERMANOVA, *p* < 0.001). Figure 6 shows a distinct clustering of the samples based on the sampling steps with an important cluster separating the orchard from the rest of the post-harvest stages. This revealed the presence of unique bacterial and fungal communities on the surface of apples picked from the orchard. For the steps following the orchard, a higher similarity was demonstrated between the different samples at these steps.

**Figure 7:**
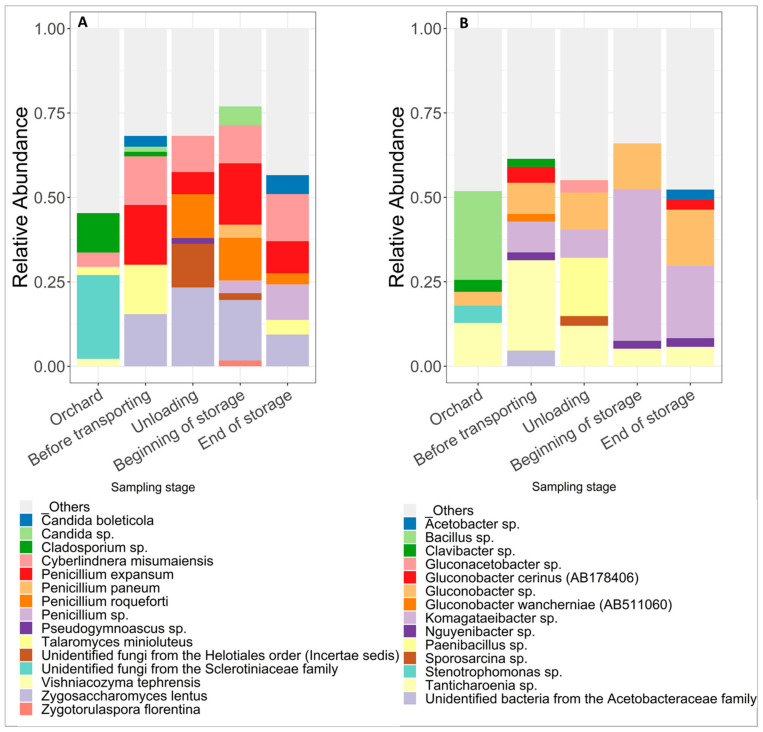
Relative abundance of fungal (**A**) and bacterial (**B**) top 10 species detected at the surface of cider-apples picked at different post-harvest sampling steps. Fungal and bacterial species with less than 0.5% are reported as “others”. When the taxonomic identification wasn’t possible to the species level, the ASV was identified by the lowest possible level of the phylogenetic tree.

The differences, between the five sampling steps, in the composition and the relative abundance (RA) of the top 10 fungal and bacterial species were shown in Figure 7 (Figure 7A,B, respectively). When the taxonomic identification wasn’t possible at the species level, the ASVs were identified using the lowest level possible in the phylogenetic tree. The species represented with less than 0.5% of the total number of reads were clustered as “Others”. The composition and RA of the fungal and bacterial ASVs were different between the orchard and the rest of the post-harvest steps. In the latter, an important similarity in the taxa was shown between the different samples with comparable RA (Figure 7).

Apples picked at the orchard step appear to feature different bacterial microbiota than those shown at the rest of the stages, especially due to the dominance in the orchard of species from the *Bacillus* genera (26.31%). The latter wasn’t abundant on the surface of apples picked from the transporting bin, unloading and storage. Species belonging to the genus *Komagataeibacter* weren’t abundant in the orchard but were remarkably abundant on the apples sampled in the transporting bin and were maintained in the rest of the sampling stages. Furthermore, species belonging to the genera *Gluconobacter* and *Tanticharoenia* were abundant across all samples. Also, *Paenibacillus* and *Sporosarcina* were abundant in the unloading stage. In the last two stages, a loss in diversity is highlighted, compared to the other stages, with the abundance of *Nguyenibacter*. Same results were illustrated for the fungal microbiota, indicating that the different sampling steps affected the fungal compositions on the surface of cider-apples.

At the orchard, the most abundant taxonomies were *Cladosporium* species belonging to the family *Sclerotiniacae*, *Cyberlindnera mismumaiensis*, *Talaromyces minioluteus* and *Vishniacozyma tephrensis*. For the remaining steps, drastic changes in the composition of the microbiome were observed. *Penicillium expansum* and *Zygosaccharomyces* appear as most abundant taxa in the transporting bin and are maintained throughout the different sampling steps. Moreover, *Penicillium paneum*, *Penicillium roqueforti* and other species belonging to the *Penicillium* genera were also abundant in the last three sampling stages. Yeasts as the genera *Zygosaccharomyces* and *Candida* were also abundant starting from the transporting bin and maintained in the storage step.

## 4. Discussion

The epiphytic microbiome of fruits consists of a large number of microorganisms who play more or less an important role in maintaining health, quality and disease resistance of the fruit during pre and post-harvest steps. So far, most microbiome studies associated with apples have mainly focused on the evaluation of the microbial diversity at a single post-harvest point. Moreover, Wassermann et al. studied the effect of hot water treatment and storage periods on the apple fungal and bacterial microbiome [27]. Shen et al. investigated epiphytic fungal community and diversity on apples of the variety “Fuji” at point of harvest [28]. Other studies carried out by Wassermann et al. and Abdelfattah et al. showed basic insights into the apple fruit microbiome highlighting the differences in bacterial and fungal composition of different apple tissues and the impact of the organic and conventional managements on the microbiota to which the consumer in directly exposed [29]. Liu et al. investigated the impact on the apple endophytic microbiota of different rootstock/scion combinations, suggesting that the latter has a possible effect on the composition of the microbial community [30]. Moreover, in a study aimed to examine the epiphytic yeast and bacteria, of five varieties of cider-apple, involved in the fermentation process, Alonso et al. highlighted the limiting factors of the DGGE technique for the characterization of the apple microbiota and suggested the use of sequencing techniques that would allow more exhaustive results [31]. However, to this day, little is known about the diversity of the epiphytic microbiome associated with apples used to produce cider. In fact, most research related to cider microbiome has concentrated on bacteria of œnological interest, like acetic and lactic acid bacteria present in the microflora in fermented cider. A study carried out by Misery et al. examined the microbiome of different ciders for distillation originating from several producers, at three main stages of the fermentation process, while focusing on phytopathogenic microorganisms and those associated with cider spoilage [32]. Nevertheless, identifying the different epiphytic microbiomes observed at various post-harvest stages could help explore indigenous antagonistic strains against *P. expansum* and therefore improve prevention practices against the blue mold disease in cider-apples.

In this study, culture-dependent and independent (using the Illumina sequencing technique) methods were combined to determine the composition of epiphytic bacterial and fungal communities present on the cider-apples sampled at the orchard and at different post-harvest steps. The combination of the two techniques is essential to have a general overview of the diversity of the apple microbiota. In fact, non-cultivable microorganisms are omitted using only culture-dependent method while the DNA studies reflect both active and non-active microorganisms. The differences in the results of the two techniques could be explained by the fact that only a small proportion (<5%) of microbes are cultivable [28]. The results of the culture-dependent method demonstrated that the transportation step seemed to have an effect on both mold and bacteria populations. However, the yeast population was more resilient to the changes occurring during the transportation and the culturable population counts were similar in the first three steps and significantly decreased in the storage silo.

The ITS and 16S rRNA sequencing technology provides deeper insight into fungal and bacterial communities [28]. The results using the metabarcoding technique revealed significant differences in bacterial and fungal population composition and diversity among the samples from the various ecosystems related to the different steps of the apple-cider production chain. Changes in the epiphytic fungal communities were tested by comparative analyses. Both alpha and beta diversity showed a highly significant difference in the composition of the fungal and bacterial communities on the surface of apples between samples picked in the orchard and those picked at the rest of the sampling steps (in the transporting bin, during unloading and in the storage silo). Moreover, the bacterial and fungal ASV numbers in the orchard were consistently higher than those in the rest of the sampling steps indicating a more diverse microbial community of the cider-apples at this step. The latter might determine a more stable and healthier ecosystem [33].

This could explain the low levels of patulin contamination in cider-apples sampled at the orchard, despite the results of qPCR illustrating the presence of *P. expansum* DNA at high amounts at the surface of those same apples (*P. expansum* was found on 88.46% of the sampled apples in the orchard). Indeed, the epiphytic ecosystem on the apples in the orchard could enable competitive interactions or antibiosis against *P. expansum*. Moreover, the plot of PCoA for the fungal and bacterial analysis demonstrated a clear clustering within all samples based on the sampling steps with two different clusters separating the orchard and the rest of the steps. Those results revealed that there was a higher similarity between the microbial communities on the surface of apples picked at the steps succeeding the mechanical harvest of apples and that the apples picked from the orchard share a more diverse fungal and bacterial community. In fact, Wisniewski et al. suggested that the fruit microbiome presents a large degree of plasticity that is displayed due to the different pre and post-harvest managements [34]. The high levels of patulin detected at the rest of the sampling steps suggested that the mechanical harvest before the loading of apples in the transporting bin could have created favorable conditions for the toxinogenesis within an altered epiphytic microbial ecosystem. However, the core microbiome of apples wasn’t considered in this study although it may present several fungal-induced infections [35]. In fact, spores of *P. expansum* present inside the seeds of apples, under favorable conditions, could penetrate through the hard seed shell to the soft vegetative tissue of the fruit, germinate and grow producing patulin [36]. Moreover, in a study aimed to identify endophytic fungal flora responsible for spoilage in fresh apples, Soliman et al. isolated five *Penicillium* spp. including *P. expansum*, that was able to produce patulin when inoculated on apple puree [9]. This indicates that potential presence of *Penicillium* spp. in the core microbiome of the analyzed cider apples could have contributed to the high levels of patulin detected throughout the different sampling steps.

Furthermore, a wide variation of the patulin content was observed between the different samples in the bin. Similar results were reported by Brown et al. who found an important variation in patulin levels in apple juice made with apples obtained from the same lot but different bins [6]. Also, Jackson et al. highlighted the heterogeneous distribution of patulin within same batches of apples while analyzing patulin levels in batches of ground-harvested cider-apples (coefficient of variation = 13.9 to 112%) [37]. These data highlight the general sampling problems associated with mycotoxin analysis and the heterogeneous distribution of the mycotoxin within the substrate. Furthermore, the sampled apples in the bin sustained shocks more or less severe due to the harvest machine explaining the high variability of patulin content in the different samples [15]. Moreover, Baert el al. explained that patulin production by *P. expansum* was susceptible to a natural high variability in apples [38]. Nevertheless, the *P. expansum* DNA abundance was steady throughout all the process stages. Indicating that although *P. expansum* was present on the surface of apples at the orchard it wasn’t able to produce patulin and the patulin accumulation was important after the mechanical harvest of apples. The mechanical harvest of apples could have caused damages to the surface of apples, facilitating the entry and colonization of wounds by *P. expansum* and therefore patulin production.

During the different sampling steps, patulin production by *P. expansum* was highly influenced by a variety of intrinsic, extrinsic and implicit factors [39]. In fact, the qualitative characteristics of the fruit can impact the severity of post-harvest pathogens [40] and physiological changes induced by the post-harvest ripening process can contribute to the interactions between pathogens and the apple tissue [41]. A study carried out by Konstantinou et al. on various apple cultivars in Greece examined the correlation between the susceptibility of the apples to *P. expansum* and the qualitative characteristics of the fruit. It was demonstrated that the susceptibility to blue mold was negatively correlated with the fruit firmness and phenol concentration and patulin production was negatively correlated with the acidity of the fruit [41]. During the post-harvest stages, apple softens by 25–50% and this is mostly due to the production of ethylene, which is a major ripening hormone in climacteric fruits such as apples [42]. Also, the temperature plays an important role in maintaining the firmness of the fruit; a temperature ranging between 0–3 °C is considered an optimal post-harvest temperature for slowing the loss of firmness. However, through the majority of the post-harvest handling chain of cider-apples, the fruits are exposed to non-optimal temperatures around 20 °C making them more susceptible to *P. expansum* growth and patulin production. Besides, Damoglou et al. revealed a decrease in the production of patulin by *P. expansum* after a period of an important patulin accumulation like it was observed in the two last sampling steps [43]. Also, Baert et al. recorded a decrease in the amount of patulin after a significant production of this mycotoxin due to an increase in the volume of the *P. expansum* rot. This decrease could have been caused by intra- or extracellular enzymes that metabolize patulin [38].

Also, the post-harvest epyphitic microbiome of apples could have an important effect on the patulin production by *P. expansum* by the intervention of microbial species that could have a competitive effect on the growth of *P. expansum* and/or effect on the toxinogenesis. Different microorganisms could have an effect on the adsorption of patulin and its biodetoxification. Indeed, the fruit surface could contain beneficial microorganisms exhibiting antagonist effects against post-harvest pathogens and therefore could be used as biocontrol agents to protect harvested fruits. Some yeast strains such as *Rhodosporidium kratochvilovae* are able to detoxify patulin by degrading it into a less toxic degradation product the desoxypatulinic acid [44,45]. Other microbial species exhibited the ability to degrade patulin such as *Saccharomyces cerevisiae*, that is capable of degrading patulin to (*E*)- and (*Z*)-ascladiol during alcoholic fermentation of apple juice for cider production [45].

In the present study, the composition and richness of the epiphytic microbiome of the cider-apples was investigated and discussed per sampling step. The results of the fungal diversity show that *Ascomycota* was the predominantly detected phylum across all samples coinciding with the results of Shen et al. and Abdelfattah et al. [28,29]. *Ascomycota* is the largest phylum of fungi kingdom with approximately 64,000 fungal species [46]. The *Eurotiales* is a large order of *Ascomycetes* and it includes 1187 species ditributed over 27 genera including *Aspergillus* and *Penicillium*, one of the most important food spoilage organisms and mycotoxin producers [47]. The second most abundant phylum detected across the samples was *Basidiomycota*, especially on the surface of apples sampled in the orchard and inside the transporting bin. The results of the most abundant fungal species identified on the surface of cider-apples in the orchard revealed the presence of yeasts that were found in the soil such as *Vishniacozyma* and *Cyberlindnera* [48,49]. The latter was detected in the soil adjacent to fruit trees from the *Rosaceae* family [49] and was maintained on the surface of the cider-apples in this study until the storage step. Moreover, *Vishniacozyma* was detected by Wassermann et al. with high abundance on the surface of healthy apples [27]. Other abundant fungi on the surface of the cider-apples at the orchard were *Sclerotiniaceae*. One of the members of the *Sclerotiniaceae* family of *Helotiales* is the genus *Monilinia*, known to be a phytopathogenic agent on pear fruits, causing brown rot and blossom blight in pome fruit [20,50]. Also, *Cladiosporium* were abundant on the surface of the apples picked from the ground at the orchard. The *Cladiosporium* genera is a post-harvest pathogen and is known to cause core browning and moldy core in apples [51]. However, antagonistic *Cladosporium* isolates were used as biocontrol agents to control apple scab caused by *Venturia inaequalis* in orchards [52]. Furthermore, *P. expansum* was not part of the most abundant taxa in the orchard, even though it was significantly found by qPCR on the surface of the apples. This could be explained by the important richness of the apple epiphytic microbiome at this step, overshadowing the presence of *P. expansum*. The latter was detected in the most abundant taxa in the apples picked inside the transporting bin and was maintained until the storage step. In fact, drastic changes in the composition of the microbiome were observed following the harvest step. *Talaromyces minioluteus* was abundant on the apples inside the transportation bin. A recent study conducted by Stošić et al. reported *Talaromyces minioluteus* as a post-harvest plant pathogen on quince, tomato, and orange fruit and onion bulbs in Serbia. Prior to this study, this species has not been considered as an important plant pathogen, even though it has been isolated from various plant hosts [53]. Another spoilage strain was abundant in the transporting bin and in the rest of the post-harvest steps: *Zygosaccharomyces* sp. Previous studies reported that the *Zygosaccharomyces* genus holds problematic yeast populations exhibiting spoilage activity in fruit juice and concentrates [54]. *Candida* was abundant in the transporting bin and in the last two sampling steps. Some species of *Candida* are known to be effective biocontrol agent used to control post-harvest diseases of fruits and vegetables. They can act as inhibitor of post-harvest pathogens [55]. Moreover, other *Candida* antagonist yeasts have direct effect on the mycotoxin patulin and are capable of degrading it and therefore ensuring detoxification of patulin in fruit-derived products [56]. Therefore, the presence of *Candida* on the surface of apples could explain the decrease in patulin levels across the different samples of apples picked at the last two steps of post-harvest. Moreover, *Pseudogymnoascus* sp. was also abundant on the surface of apples after the harvest. It has been detected by Shen et al. on the surface of cold stored apples of the variety ‘Fuji’ [28]. Different species of *Penicillium* were abundant on the surface of the apples picked at the four steps of post-harvest. They could be potential competitors to *P. expansum* growth. A study carried out by Frisvad in 2018 highlighted the presence of 22 species of *Penillium* producers of patulin. Of which, *Penicillium paneum* produced patulin in silage due to its ability to grow in substrates with a high concentration of acetic acid [57]. This species is abundant in our study in the last three sampling stages. The high levels of patulin detected in these steps could also be explained by the presence of this species on the surface of the sampled cider-apples.

Furthermore, the bacterial microbiome on the surface of apples picked at the orchard was rich with indigenous soil bacteria that contaminated the apples when they were shaken to the ground before harvesting. In fact, *Bacillus* sp. was only abundant on the surface of the ground harvested apples. Many studies highlighted different strains of *Bacillus* that have been isolated from apple rhizosphere [58,59,60]. Moreover, *Bacillus* sp. strains could exhibit antifungal activity against the growth of pathogenic fungi. Ju et al. identified and evaluated a *Bacillus* sp. strain as a potential biocontrol agent against *Fusarium* sp. in apple seedlings [60]. The genera *Stenotrophomonas* and *Gluconobacter* were also abundant on the surface of apples in the orchard. They were isolated from bacterial communities within the soil ecosystem [61,62]. They both exhibited biocontrol activity against fruit rots. *Stenotrophomonas* was active against *Fusarium proliferatum* species in muskmelon [62] and *Gluconobacter* strains, naturally present on the surface of apples, showed antifungal and anti-patulin activity against *P. expansum* species [63]. Moreover, important changes in the composition of the bacterial microbiome were also observed following the harvest step, the microbiome contained mainly bacteria traditionally present in the final product of apple-cider. *Komagataeibacter* became predominantly present in the transporting bin and was maintained on the surface of the apples until the storage silo. *Komagataeibacter* species were isolated from apple-cider vinegar [64]. Also, *Paenibacillus* were abundantly present on the surface of apples picked at the post-harvest step of unloading. It is a plant growth-promoting rhizobacteria (PGPR) that also showed promising biocontrol effect on post-harvest pathogens of apple fruits [65]. In the last two sampling steps, the bacterial community seems to be less diverse with an abundant occurrence of *Nguyenibacter* and *Acetobacter*. In fact, *Acetobacter* sp. is an acetic acid bacteria (AAB) associated to malolactic fermentation and it was detected by Misery et al. at the end of the alcoholic fermentation and the maturation period of different ciders [32]. The abundance of *Acetobacter* in the storage stage could have been enhanced due to the ethanol produced during storage by the indigenous yeasts.

These results highlighted the presence of important indigenous microbiota that could exhibit antifungal and anti-patulin activity against the growth and the toxigenicity of *P. expansum*. Thus, it could be interesting to harness these species for biological control approaches to develop promising and sustainable future strategies to prevent post-harvest decay of fresh and stored produce. These strategies could replace the conventional control of the post-harvest blue mold decay by the use of chemical fungicides [66]. However, their safe usage should be further investigated because of the unspecific degrading mechanism(s) of patulin, in addition to the incomplete toxicological assessment of degradation by-products [66].

## Figures and Tables

**Table 1:** List of primers used in this study.

Primer	Sequence (5′-3′)
Pexp_patF_F	ATGAAATCCTCCCTGTGGGTTAGT
Pexp_patF_R	GAAGGATAATTTCCGGGGTAGTCATT
ITS86F	GTGAATCATCGAATCTTTGAA
ITS4R	TCCTCCGCTTATTGATATGC
341F	CCTACGGGNGGCWGCAG ^a^
785R	GACTACHVGGGTATCTAATCC

^a^ A “W” represents a nucleotide that could be either an A or a T; a “H” represents a nucleotide that could be either an A or a C or a T; a “V” represents a nucleotide that could be either an A or a C or a G and “N” represents any nucleotide.

**Table 2:** Patulin content in apple samples collected at different steps of the apple cider production chain.

Sampling Step	n ^1^	Percentage of Positive Samples ^2^	Patulin Content Range ^3^ (µg·kg^−1^ of Apple)	Tukey Grouping ^4^
Orchard	16	6	0–280	a
Before transporting	19	47	0–2943	b
Unloading	19	95	0–1169	ab
Beginning of storage	13	69	0–909	ab

^1^ Number of analyzed samples (Orchard: 15 individual apples and one group of five apples; Before transporting: 15 individual apples, three groups of five apples and one group of 15 apples; Unloading: 15 individual apples, three groups of five apples and one group of 15 apples; Beginning of storage: 12 individual apples, one group of five apples), total number of apples analyzed = 127. ^2^ The percentage of samples (including individual apples, groups of five apples and groups of 15 apples) exhibiting patulin contamination. ^3^ The lowest and highest patulin concentration reported across all samples. ^4^ Different groups revealed by post-hoc Tukey’s HSD test, *p* < 0.05.

**Table 3:** DNA amounts of *P. expansum* at the surface of cider-apples picked at different post-harvest steps.

Sampling Steps	n ^1^	Percentage of Positive Samples	Mean ± SE ^2^ (Log DNA *P. expansum*/g of Apple)
Orchard	26	88.46	4.21 ± 2.0
Before transporting	19	84.21	4.26 ± 2.2
Unloading	19	84.21	3.99 ± 2.3
Beginning of storage	19	84.21	4.25 ± 2.4
End of storage	19	84.21	4.09 ± 2.5

^1^ Number of analyzed samples (Orchard: 25 individual apples, one group of five apples and one group of 15 apples; the rest of the sampling steps: 15 individual apples, three groups of five apples and one group of 15 apples), total number of apples analyzed = 225. ^2^ Mean of *P. expansum* DNA amounts across all the samples; SE: Standard Error.

## Data Availability

The data presented in this study are available on request from the corresponding authors.

## Abbreviations

*P. expansum*	*Penicillium expansum*
IARC	International Agency for Research on Cancer
EU	European Union
CFU	Colony-forming unit
ITS	Internal transcribed spacer
ASV	Amplicon sequence variant
RA	Relative abundance
PGPR	Plant growth promoting rhizobacteria
AAB	Acetic acid bacteria
